# Can pyraclostrobin and epoxiconazole protect conventional and stay-green maize varieties grown under drought stress?

**DOI:** 10.1371/journal.pone.0221116

**Published:** 2019-08-20

**Authors:** Hanna Sulewska, Karolina Ratajczak, Katarzyna Panasiewicz, Hazem M. Kalaji

**Affiliations:** 1 Department of Agronomy, Faculty of Agronomy and Bioengineering, Poznan University of Life Sciences, Poznan, Poland; 2 Department of Plant Physiology, Faculty of Agriculture and Biology, Warsaw University of Life Sciences WULS-SGGW, Warsaw, Poland; United Arab Emirates University, UNITED ARAB EMIRATES

## Abstract

Risks associated with drought are increasing and are a global problem. Therefore, there is a need for new solutions for the safe production of food, while maintaining respect for the environment. Fungicides are designed to protect maize plants against disease, but some of the active substances used in preparations can also promote plant growth, which is known as the ‘physiological effect’. However, there is a paucity of information as to how some of the ‘new generation’ fungicides act in stimulating grain yield in plants under abiotic stress, especially drought. Therefore, the effects of these products on conventional and stay-green maize varieties need to be better understood in order to reduce losses caused by droughts and to maximize production. In this study, the effect of a pyraclostrobin + epoxiconazole fungicide preparation on maize plants was evaluated at different times after spraying; during induced drought conditions and again during the regeneration process of the plants. The preparation was applied to ‘KWS 1325’ (conventional) and ‘Ambrosini’ (stay-green) varieties at the recommended dose, three times in greenhouse conditions. Plant gas exchange, plant water use efficiency, chlorophyll fluorescence and fresh and dry plant biomass were evaluated.

The pyraclostrobin + epoxiconazole preparation increased stomatal conductance and photosynthesis intensity in the ‘Ambrosini’ plants. When maintained under a high light intensity, the variety used increased efficiency and exchanged excessive energy in the form of thermal energy to protect the maize leaf from light-induced damage under drought stress.

Plant photosynthetic efficiency (ETR and Yield parameters) during drought stress and after regeneration was significantly higher in treated plants than in the controls. Thus, the beneficial effects on the physiology of the maize varieties grown under drought stress from the fungicide application are significant for farmers and growers.

## Introduction

Climatologists predict that over the next decades some areas will experience both an increase in the occurrence of drought and an escalation of flooding incidence [1]. Drought has been assessed as one of the major reasons for crop failure, reducing global average crop yields by 50% or more [2]. Some research has shown that yield reduction varies widely depending on species, with higher yield reduction in maize (39.3%) compared to wheat (20.6%), at approximately 40% water reduction [3]. In recent years, the extent of damage caused by drought has increased, which tends to occur more frequently, and is especially unfavourable for maize production. For decades, plant breeders of strategic species have worked on this problem, and have introduced new plant varieties that are less susceptible to drought. However, the latter process (tolerance achievement through traditional breeding methods) takes a long time and is problematic because the sources of resistivity are not currently available [4].

One possibility to deal with the effect of drought stress is to reduce the negative impact of water deficiency through the use of biostimulators, which support plant tolerance during stress in the vegetative period. However, it is difficult to predict the exact moment that drought will occur, so effective and long-acting preparations have been sought by crop growers. Another solution to protect plants during drought is the application of polymers. Such molecules absorb water when it is available and release it to the plants during periods of limited precipitation. However, this method is difficult to apply on a large scale due to economic reasons. Nevertheless, the best solution could be a combination of fungicide protection with actions that stimulate plant growth. With cognisance that the main function of agriculture is to feed a growing number of people on the planet, research should be focused on the reduction of plant response to various types of environmental stress.

Currently, abiotic stress still limits plant yield even in the most technologically advanced farms. A reduction in abiotic stress is often very difficult to achieve and biostimulators could play a crucial role in this regard. In order to reduce costs, chemical companies register and introduce market preparations that are often based on insignificant, inexpensive and naturally occurring components. The preparations are described as remedies for all problems, as they support plant growth and may also be used in organic farming [5]. Some studies indicate that fungicides can improve physiological traits, such as photosynthesis, the antioxidant system, nitrogen metabolism (and thus promote growth) [6] and increase grain yield, regardless of whether the plant is affected by a disease [7,8].

The use of biostimulators has been shown to reduce crop yield loss and improve both crop yield and quality. It has been shown in many studies that biostimulators are able to alleviate the negative effects caused by climatic stress factors. Such factors include extreme temperature and drought, or chemical factors, such as salinity or even environmental pollution [9,10].

Plant reaction to aridity is exhibited in many physiological and biochemical processes, such as stomatal closure, reduced transpiration rates, accumulation of abscisic acid or formation of radical scavenging compounds, which result in growth inhibition and decreased photosynthetic rates, as well as the synthesis of new proteins and mRNAs [11].

Currently, it has been shown that some biostimulators can protect plants from stress by increasing the energy production of plants, accelerating cytoplasm flow in cells and tissues, stabilising the products of biochemical reactions, and maximising the effectiveness of natural hormone synthesis [12,13]. However, the effect may be time-limited. In the literature, some studies document a positive plant response with only slight side effects, or even the lack of any such reaction. When there is a short period between the application of a particular substance and the occurrence of stress, it is often connected to a positive response from the plants. Yet, when it is applied a long time after negative environmental conditions, a slight effect, or even a lack of effect may be observed [14]. Indeed, in some cases, negative effects are also observed [15,16]. The most beneficial effect would be induced resistance from the plants. For example, the Harpin (Ea) protein has been shown to induce plant resistance, while a new generation of broad spectrum fungicides, such as strobilurin has been shown to enhance plant growth and biomass production. Their action delays leaf senescence, increases nitrate uptake, regulates phytohormonal levels to overcome stress and alleviates oxidative plant stress [17, 18]. Triazoles promote responses by pre-inducing resistance to abiotic stresses, such as drought [19], and the effect of substances from this group may be inhibitory or stimulatory as it depends on the compound, concentration used and type of plant [20].

Most plant species, including maize, can defend themselves, adapt to adverse conditions, at least in part to avoid the impact of a stressor. Often, however, a defensive response comes too late, and it is difficult to replace yield losses. Therefore, it may be advisable to support plants with an application of a specific restorative preparation. If used when the plants are healthy, the biostimulators could change the metabolism in such a way that the plants are strengthened, thereby making them more resistant to pathogen attack or drought.

The plant development phase of maize is strictly dependent on the reaction of plants to drought stress. Commonly, it has been shown that it is more difficult for maize to survive stress that occurs in late developmental stages (pollination and the grain filling stage), and which is associated with major losses of crop yield. During generative development, a lack of water can affect yield components. Studies have documented strongly reduced kernel numbers in maize plants, where approximately 40% of the final ear length was reached at silking and the remaining 60% was attained after silking [21]. According to NeSmith and Ritchie [22], 37 days of water deficit can cause barrenness in 78% of plants during significant phases, such as tassel emergence and silking, while kernel number in ear-bearing plants could be reduced by 75% compared with plants grown without water deficit. Early reaction to water stress is important as plants build the first line of defence during this time, while it is harder for plants to survive more persistent stress periods. In this case, plants need to undergo an acclimation process [23]. Plants need to change their metabolic processes or structures, which results from changes in regulation of gene expression. To prevent serious tissue damage caused by environmental stress factors, plants need to activate physiological responses [24].

The aim of the experiment was to evaluate the biostimulatory activity of the fungicide pyraclostrobin (500 F) in combination with epoxiconazole on two maize varieties, i.e., conventional and stay-green, when subjected to drought stress.

## Materials and methods

### Experiment description

Two-factorial pot experiments were carried out three times (i.e. Run 1–3) in the greenhouse of the Department of Agronomy at the University of Life Sciences in Poznan in 2013–2014 (Table 1). A randomized block method was used.

**Table 1:** Timesheet of experiment.

Series of experiments	Sowing date	Plant thinning date	Initial measurement date (before inducing stress) - 0 days	Plant collection date after regeneration (49 days after the start of measurement)
Run 1	13/08/13	19/08/13	27/10/13	15/12/13
Run 2	04/09/13	11/09/13	10/11/13	29/12/13
Run 3	07/02/14	14/02/14	28/03/14	16/05/14

Two varieties of maize were subjected to drought stress. In all sets of experiments, each object under study was analysed in four replications. The first-order factor was the variety of the maize plant: ‘KWS 1325’ (conventional) and ‘Ambrosini’ (stay-green). The second-order factor was the use of a preparation: (133 g / L) pyraclostrobin (F 500) together with (50 g / L) epoxiconazole suspo-emulsion (SE) (1.5 L / ha) foliar spray application and pure control (without preparation).

In the period from sowing to the start of induced drought, the maize plants were maintained in optimal conditions. They were watered regularly (350 mL H_2_O / pot per 72 h) and double-fertilised with Florovit (350 mL of solution in a concentration of 5 mL Florovit /1 L H_2_O). A biostimulant was used at the 11/12 leaf stage on the second day after the start of measurements and was applied as a single foliar spraying. The application of the preparation was made in a working fluid concentration of 5 mL / L H_2_O (1 L / 200 L H_2_O / ha) using a laboratory sprayer. The sprayer was fitted with a set of Tee Jet flat spray nozzles (type DGTJ60 11003) with an output of 305 L / ha at an operating pressure of 0.35 MPa. Control plants were sprayed with distilled water at the same time and at the same dose. Drought stress was induced in the 12/13 leaf stage on the third day after spraying (5 days from the start of the measurement). The plants had not been watered for 23 days and after showing severe symptoms of drought (leaf curl maintained 24 hours a day), measurements of their physiological status were performed. The soil water content was reduced to 8–6% (w/w, pF = 3.2); a level barely accessible by the plants, but not exceeding the permanent wilting point. Then, in order to trace their regeneration, the plants were irrigated again in the same way as at the beginning of the experiment. The regeneration of plants after undergoing drought stress was assessed after 20 days of irrigation (48 days from the start of measurement).

The conditions in the greenhouse while conducting the experiments were as follows: photoperiod- 16 h light / 8 h dark; temperature- 25–30°C. “Universal soil” (bought in a garden centre), consisting of 0.47 g phosphorus pentoxide (P_2_O_5_) / kg soil, 0.1 g potassium oxide (K_2_O) / kg soil, 0.81 g magnesium (Mg) / kg at soil pH 6.4 in 1M potassium chloride (KCl).

Each of the 5 L pots was filled with 6 kg of slightly firm soil. Then, 5 maize kernels were laid on the soil and covered with a new layer of soil. As a result, the plants germinated at the same time. A similar plant was left in each pot after thinning. All measurements were conducted for each of the plants on the same day and the order of replication was maintained. Measurements of the physiological status of the plants were always performed on the 11th leaf of the plants. All parameters were measured from 09:00 until 18:00 in an environmentally controlled greenhouse.

Before harvesting, plant height was measured from the surface of the pot to the tip of the longest leaf. After harvesting, the plants were weighed, and their fresh and dry biomass were recorded (at 105°C).

The results were statistically analysed with Statistica 10 software. Analysis of variance was used and the significance of difference was assessed with a Tukey’s test (p ≤ 0.05). The statistical model was used as follows:

$$\begin{array}{l} Y_{ijklm=m+R_{i}+RR_{j}(R_{i})+V_{k}+S_{l}+V\times S_{kl}+e_{ijklm}} \end{array}$$ (1)

where:

Y_ijklm_−observation value

m–general population mean

R_i_−effect of run i (i = 1,2,3)

RR_j_−effect of replication j within run i (j = 1,2,3,4)

V_k_−effect of variety k (k = 1,2)

S_l_−effect of spraying l (l = 1,2)

VxS_kl_−interaction effect of variety by spraying

e_ijklm_−random error term

Experimental variant heatmap with cluster analyses were performed to analyse similarities between plant photosynthesis and chlorophyll fluorescence parameters. Euclidean distance measures and Ward hierarchical clustering were used to determine the dendrogram.

### Plant materials

Two maize (*Zea mays* L.) hybrids, traditional leaf senenscence ‘KWS 1325’ and stay-green ‘Ambrosini’ were obtained from KWS-Poland for experiments. Single- cross hybrid ‘KWS 1325’ with the class of earliness FAO 230 is suitable for grain, according to Polish post-registration Research Centre for Cultivar Testing experiments (2011–2014) yielded 12.1 t/ha of dry grain (14% H_2_O) with moisture content during harvesting 26.2%. Triple-cross hybrid ‘Ambrosini’ with the class of earliness FAO 220 is suitable for silage and grain, in 2011–2014 yielded 11.8 t/ha of grain (14% H_2_O), and moisture content during harvesting 25.9%.

### Assessment of plant physiological status

#### Plant gas exchange

Photosynthetic rate (A) of single leaves was measured on the first fully mature leaf during the elongation stage using a portable photosynthesis system LCpro-SD (ADC BioScientific Ltd., UK) with a narrow leaf chamber (area: 5.8 cm^2^). The carbon dioxide (CO_2_) concentration (reference CO_2_) in the leaf chamber was maintained at 360 ppm and the leaf chamber temperature at 25±1°C. The flow rate of air was approximately 200 μmol s^-1^. The remaining settings (e.g. Reference H_2_O) were kept as ambient conditions. Formula (1) was used to determine plant water use efficiency (WUE):

$$\begin{array}{l} WUE=\frac{A}{E} \end{array}$$ (2)

where A represents photosynthetic rate (μmol CO_2_ m^-2^ s^-1^) and E represents transpiration rate (mmol H_2_O m^-2^ s^-1^).

During the measurements to construct light response curves, photosynthetic photon flux density (PPFD) was 1500, 1000, 700, 400, 200, 100, 50, and 0 μmol m^-2^ s^-1^, adjusted automatically by a red-blue light-emitting diode (LED) light source (LCP Narrow Lamp, ADC BioScientific Ltd., UK). The maximum photosynthetic rate (Pmax), irradiance compensation point (Ec), saturating irradiance (Ek), half saturation (Km) and dark respiration (R) were calculated on the basis of the light-response curves (P-E curve),

P_E_ = (P_max_*E_λ_) /(K_m_+E_λ_), where P_E_ is the photosynthetic rate at any irradiance E, E_λ_ is the spectral irradiance (in μmol m^-2^ s^-1^), and K_m_ is the half saturation constant (E_k_) when P_E_ = P_max_/2.

$$P_{E}=\frac{P_{max}\left(E_{\lambda }-E_{c}\right)}{K_{m}+\left(E_{\lambda }-E_{c}\right)}$$ (3)

The parameters describing the function shown above were determined by minimising the sum of squares of errors.

### Chlorophyll fluorescence measurements

Chlorophyll fluorescence was measured on the same leaf as used to determine photosynthesis with a Multi-Mode Chlorophyll Fluorometer OS5p (OPTI-SCIENCES.INC., Hudson, USA) with Photosynthetic Active Radiation (PAR) Clip (allows for the measurement of PAR or PPFD and leaf temperature along with the Yield test).

The OS5p Modulated Fluorometer is a multipurpose portable instrument designed to precisely measure chlorophyll fluorescence (OS5p User’s Guide). Settings for the fluorometer protocols were selected in accordance with the manufacturer’s instructions (OS5p User Guide). In the test, the source of modulated red light (660 nm) was used. It is important that the intensity of the measuring light is set sufficiently high to induce a fluorescence signal appropriate for photosynthetic yield measurements in light adapted samples. Modulation Intensity in the Fv/Fm Protocol was set at position 10 and in the Yield Protocol at position 17 (i.e. the factory setting for an approximate value of 0.1 μmol m^-2^ s^-1^). The gain setting in the Fv/Fm Protocol was set at position 2 and at 5 in the Yield Protocol. The saturation source was a 35W halogen lamp. The saturation pulse is a short pulse of intense light to fully reduce Photosystem II (PSII) in a leaf. The Saturation Flash Intensity values were set at Position 24 in both Protocols. The 32 value equates to approximately 15000 μmol photons m^-2^ s^-1^ with a dark clip and 8550 μmol photons with a PAR Clip. An actinic source is a light source that drives photosynthesis. For the Yield Protocol, sunlight was measured with PAR Clips at a PPFD ratio average 60 μmol photons m^-2^ s^-1^.

The following parameters were measured or/and calculated:

- **Fv/Fm** is a widely used parameter that shows the maximum quantum efficiency of PSII. This parameter is generally considered to be a ready indicator of plant photosynthetic performance; healthy samples typically achieve a maximum Fv/Fm value of approximately 0.83–0.85. Values less than this indicate that a sample has been exposed to some type of biotic or abiotic stress factor, reducing the capacity for photochemical quenching of energy within PSII [25]. Leaves were dark-adapted (with dark clips) for 30 minutes prior to the measurement of minimal fluorescence (F_0_) and maximum fluorescence (Fm). Based on these two measurements, variable fluorescence (Fv) and the maximum photochemical efficiency of PSII were then calculated (F_v_/F_m_ = Fm-F_0_/Fm).
- **Yield (Y)–**a light adapted constant-state photosynthetic test that provides a measure of the proportion of light used in photochemistry in PSII versus the amount of light absorbed by the chlorophyll associated with PSII [26]. The samples were light adapted for ca. 30 mins before Y and the relative Electron Transport Rate (ETR) were measured [27].
- **ETR**–is a parameter that is measured with a PAR Clip and is a relative measurement that provides comparative electron transport rates for PSII at different light or irradiation levels. The equation for the ETR calculation is: ETR = Y x 0.84 x 0.50 x PPFD [27].

## Results

In general, the variation in activity of the photosynthetic apparatus resulted from the two tested factors (the preparation, and varietal differences). Changes in plant photosynthetic efficiency (estimated on the basis of chlorophyll fluorescence measurements) indicated that the plants showed a balanced energy expenditure strategy after the application of the fungicide preparation.

### Effect of pyraclostrobin and epoxiconazole on yield and ETR parameters

**Fig 1:**
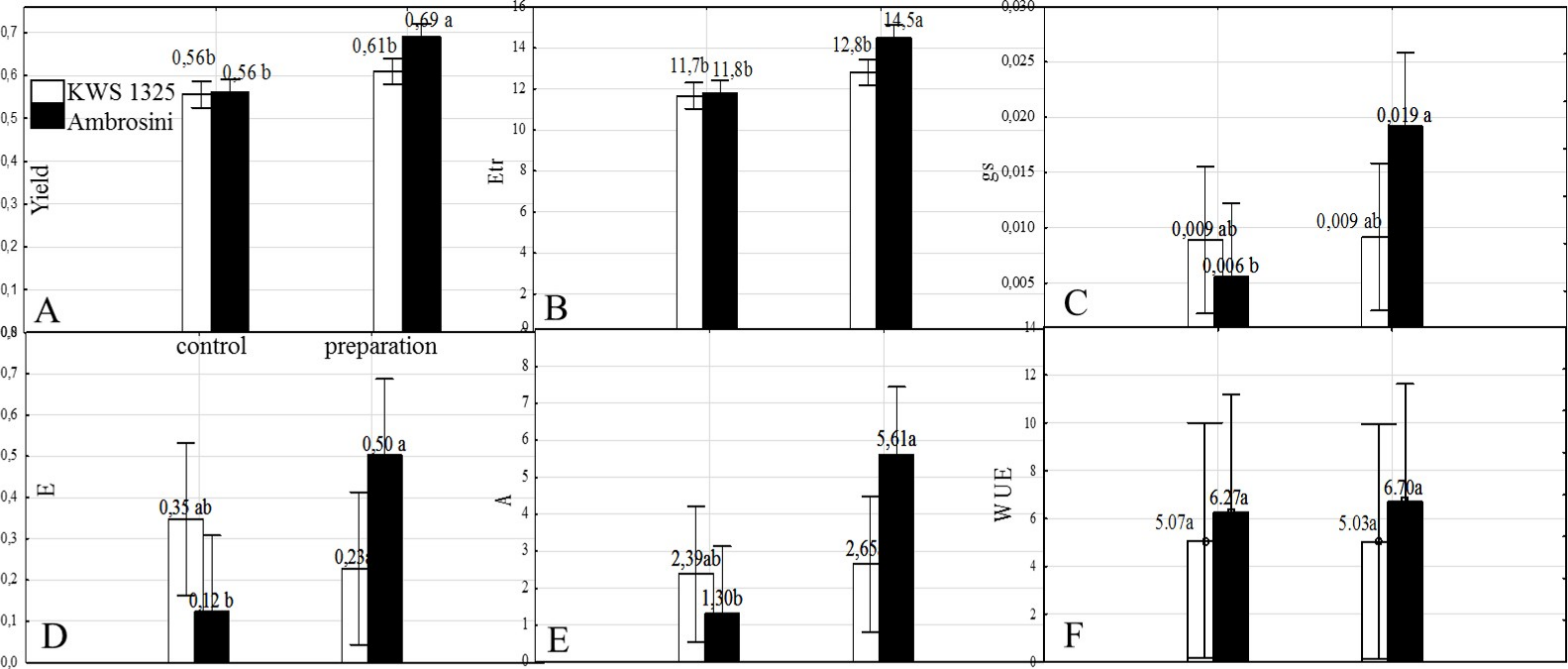
Parameters: A- Quantum Yield of photosystem II (Yield), B- Electron Transport Rate (ETR), C- Stomatal conductance of H_2_O (gs; mmol m^-2^ s^-1^), D- Transpiration rate (E; mmol m^-2^ s^-1^ at a light intensity of 400 μmol photon m^-2^ s^-1^), E- Photosynthetic rate (A; μmol CO_2_ m^-2^ s^-1^ at a light intensity of 400 μmol photon m^-2^ s^-1^), F- water use efficiency (WUE; evaluated during drought stress in plants of both varieties, depending on the preparation tested). a; b–homogeneous groups (Tukey’s test).

During the initial measurement, the stay-green ‘Ambrosini’ plant showed a significantly higher quantum yield of photochemical reaction (Yield) and ETR values than the ‘KWS 1325’ variety (Table 2, Fig 1). The preparation reduced the varietal differences between plants. The differences between the varieties were statistically insignificant (p = 0.5828) until the plants were introduced into a state of stress. Under drought conditions, ‘Ambrosini’ functioned significantly better than ‘KWS 1325’; proven by the higher Yield and ETR parameter values (Fig 1A and 1B). The yield of PSII (Yield parameter) and ETR were 13.1 and 13.3% respectively higher in the ‘Ambrosini’ than in the ‘KWS 1325’ plants treated with pyraclostrobin and epoxiconazole. After a period of regeneration, the varietal differences expressed by Yield and ETR were statistically insignificant (p = 0.8577) (Table 2). In ‘KWS 1325’, those parameters tended to remain higher than values observed before the start of the experiment. The observations made on the ‘Ambrosini’ variety were different; as this variety exhibited higher Yield and ETR parameter values than ‘KWS 1325’. The application of the preparation caused an increase in the Yield parameter value, which was observed on the third day after application. Importantly, the ETR parameter value increased on the fifth day after application and the observed response was independent of plant variety. The observed positive reaction of plants after the use of the preparation continued until the end of the experiments (Table 2).

**Table 2:** Quantum Yield of photosystem II (PSII), Electron Transport Rate (ETR) and Chlorophyll Content Index (CCI) of leaves depending on the variety and the use of the preparation.

Specification		Number of days				
		0 first measurement	2 spraying	5 stop watering	28 drought stress	48 regeneration
Quantum Yield of PSII						
Variety	KWS 1325	0.6265 ^b^	0.6757 ^a^	0.6943 ^a^	0.5830 ^b^	0.6462 ^a^
	Ambrosini	0.6559 ^a^	0.6832 ^a^	0.6851 ^a^	0.6258 ^a^	0.6508 ^a^
p-value		0.0101*	0.0749 ^ns^	0.5828 ^ns^	0.0067**	0.8577 ^ns^
Spraying	control	0.6410 ^a^	0.6789 ^a^	0.6831 ^a^	0.5582 ^b^	0.6161 ^b^
	preparation	0.6414 ^a^	0.6800 ^a^	0.6962 ^a^	0.6505 ^a^	0.6809 ^a^
p-value		0.9724 ^ns^	0.0584 ^ns^	0.4341 ^ns^	0.0000**	0.0156*
Electron Transport Rate						
Variety	KWS 1325	13.2 ^b^	14.2 ^a^	14.6 ^a^	12.2 ^b^	13.6 ^a^
	Ambrosini	13.8 ^a^	14.3 ^a^	14.4 ^a^	13.1 ^a^	13.7 ^a^
p-value		0,0109*	0.4292 ^ns^	0.5828 ^ns^	0.0067**	0.8577 ^ns^
Spraying	control	13.5 ^a^	14.3 ^a^	14.3 ^a^	11.7 ^b^	12.9 ^b^
	preparation	13.5 ^a^	14.3 ^a^	14.6 ^a^	13.7 ^a^	14.3 ^a^
p-value		0.9724 ^ns^	0.9035 ^ns^	0.4341 ^ns^	0.0000**	0.0156*
Chlorophyll Content Index						
Variety	KWS 1325	14.91 ^a^	13.59 ^a^	15.14 ^a^	15.44 ^b^	19.32 ^b^
	Ambrosini	16.05 ^a^	15.25 ^a^	17.10 ^a^	18.75 ^a^	24.52 ^a^
p-value		0.1058^ns^	0.0764^ns^	0.1494^ns^	0.0001**	0.0001**
Spraying	control	15.53 ^a^	13.62 ^a^	15.26 ^a^	15.96 ^b^	21.28 ^b^
	preparation	15.43 ^a^	15.22 ^a^	16.97 ^a^	18.24 ^a^	22.57 ^a^
p-value		0.8854^ns^	0.0864^ns^	0.2078^ns^	0.0016**	0.0400*

* statistically significant differences (*p* < 0.05)

** highly statistically significant differences (*p* < 0.01)

^ns^ no statistically significant influence on tested trait (*p* > 0.05).

Mean values that do not differ significantly have the same letter

a; b–homogeneous groups (Tukey’s test).

### Effect of pyraclostrobin and epoxiconazole on stomatal conductance, plant gas exchange, transpiration, photosynthetic rate and water use efficiency

The applied preparation caused a strong reaction on the stomata of the ‘Ambrosini’ plants. Similar reactions were not observed in the ‘KWS 1325’ plants. Stomatal conductance of ‘Ambrosini’ plants growing under drought stress and treated with the preparation was increased more than threefold (from 0.006 to 0.019) in comparison with the control group. Thus, this would indicate that during drought stress, gas exchange in these plants is more intense than in the control plants of the same variety (Fig 1C).Transpiration and photosynthetic rate of ‘Ambrosini’ treated with pyraclostrobin and epoxiconazole increased 0.38 and 4.31 respectively, in comparison to control without preparation. In ‘KWS 1325’ variety this difference in photosynthetic rate was smaller and amounted 0.26, while photosynthetic rate decreased after preparation applied (Fig 1D and 1E). Water use efficiency (WUE) was 0.43 higher in the ‘Ambrosini’ plants after preparation applied,. In ‘KWS 1325’ the same parameter slightly decreased by 0.04, although the observed differences were not statistically significant (Fig 1F).

### Effect of pyraclostrobin and epoxiconazole on Chlorophyll Content Index

Chlorophyll Content Index (CCI) in the leaves of ‘Ambrosini’ was greater than in the leaves of ‘KWS 1325’ (Table 2). In the first measurement, the CCI value of the ‘Ambrosini’ leaves was 7.6% higher (16.05) than in the ‘KWS 1325’ (14.91) leaves. Regeneration after drought stress increased the difference by up to 26.9%. The application of the preparation stimulated synthesis of chlorophyll in the leaves. The sprayed plants contained 14.3% (drought stress) and 6.1% (after regeneration) more chlorophyll than the control plants (Table 2).

### Effect of pyraclostrobin and epoxiconazole on plant morphology

The application of the preparation to the ‘KWS 1325’ plants led to an increase in the height of the plants. The difference was 10.2 cm in comparison with the control plants and was statistically significant. The height of ‘Ambrosini’ plants (both in the control and treatment) was greater than the height of the ‘KWS 1325’ plants. However, the reaction of ‘Ambrosini’ plants to the use of the preparation was weaker. The increase in height was 5.3 cm, but was only indicative of a trend. ‘Ambrosini’ is a variety where the processes of mitochondrial respiration (parameter R) during drought stress tend to be more intense than in ‘KWS 1325’ plants (Table 3). In both varieties, the use of the preparation boosted the respiratory processes significantly. Notably, the R parameter for the preparation-treated ‘Ambrosini’ plants during drought stress was significantly higher than the value of this parameter for the ‘KWS 1325’ plants and could explain the minor increase observed in dry matter of ‘Ambrosini’ plants, as well as the smaller number of maize cob buds. This was assessed after application of the preparation and regeneration of the ‘Ambrosini’ plants. In both varieties, a significantly higher R parameter value was maintained even after the plants had regenerated (Table 3). However, differences in the R parameter value were not observed between the two plant varieties.

**Table 3:** Parameter R (dark respiration), light saturation point (Ek) and light compensation point (Ec) values rated under drought stress and regeneration of the plants (μmol m^-2^ s^-1^).

Variety	Spraying	23 days after spraying (drought stress)			48 days after spraying (regeneration)		
		R	Ek	Ec	R	Ek	Ec
KWS 1325	control	0.828 ^c^	1055.3 ^a^	220.1 ^a^^b^	2.196 ^b^	764.4 ^c^	62.5 ^a^
	preparation	1.143 ^b^	991.2 ^a^^b^	77.4 ^b^^c^	2.605 ^a^	900.3 ^b^	48.3 ^c^
Ambrosini	control	0.911 ^b^^c^	917.2 ^b^^c^	289.9 ^a^	2.014 ^b^	927.4 ^b^	53.1 ^b^
	preparation	1.878 ^a^	782.2 ^c^	56.6 ^c^	2.536 ^a^	1004.8 ^a^	51.3 ^b^^c^

a; b, c–homogeneous groups (Tukey’s test)

### Effect of pyraclostrobin and epoxiconazole on light saturation point and light compensation point

Our results showed that during drought stress the ‘Ambrosini’ plants used low-intensity light less efficiently than the ‘KWS 1325’ plants (Table 3). This was shown by a significantly lower Ek value. Facilitation of plant growth through the use of the preparation produced a lower Ek value; the values decreased by 64.1 and 135.0 μmol m^-2^ s^-1^ for ‘KWS 1325’ and ‘Ambrosini’ plants, respectively. In comparison to the ‘KWS 1325’ plants, the Ek values for ‘Ambrosini’ were significantly higher in both the control and preparation-treated groups. The application of the preparation led to a higher Ek value observed after the regeneration of plants (Table 3). This was true for both the ‘Ambrosini’ and ‘KWS 1325’ plants. During drought stress, the Ec value for the ‘Ambrosini’ variety in the control group was 69.8 μmol m^-2^ s^-1^ higher than in the ‘KWS 1325’ plants. However, this difference was not statistically confirmed. This parameter shows that under drought stress ‘Ambrosini’ plants respired more intensively than ‘KWS 1325’ plants. After plant regeneration, the Ec parameter decreased in both varieties. The use of the preparation resulted in a decrease in the Ec value in the treated plants, indicating a slowdown in plant respiration. The decrease was significant for the ‘KWS 1325’ variety (14.2 μmol m^-2^ s^-1^) but insignificant for the ‘Ambrosini’ variety (1.8 μmol m^-2^ s^-1^).

### Effect of pyraclostrobin and epoxiconazole on plant dry mass

Growth of the plants was evaluated by the yield of dry matter collected after the completion of the regeneration phase (Table 4). Under control conditions, the regenerated ‘Ambrosini’ plants produced 1.12 g (4.9%) more dry matter and 2.48 g (51.6%) more maize cob buds than the ‘KWS 1325’ plants. After spraying with the preparation, the ‘Ambrosini’ variety showed a significantly higher level of dry matter yield of maize cob buds (1.04 g per plant). The ‘Ambrosini’ variety displayed a lower yield of leaves and stems.

**Table 4:** Plant dry weight (g) and height (cm) after regeneration.

Variety	Spraying	Dry weight					Plant height
		leaves	stem	panicle	cob bud	Whole plant	
KWS 1325	control	7.27^a^	10.12^a^	0.53^a^	4.81^a^	22.74^a^	126.7^b^
	preparation	7.37^a^	10.81^a^	0.54^a^	5.74^a^	24.45^a^	136.9^a^
Ambrosini	control	6.65^a^	9.42^a^	0.50^a^	7.29^b^	23.86^a^	133.6^a^
	preparation	6.54^a^	9.39^a^	0.56^a^	8.33^a^	24.74^a^	138.9^a^

a; b–homogeneous groups (Tukey’s test)

For ‘KWS 1325’, the use of the preparation led to an increase in dry biomass of a single plant (an increase of 1.71 g per plant) and in the individual elements of its structure. Moreover, the preparation stimulated the growth of plants of both varieties.

**Fig 2:**
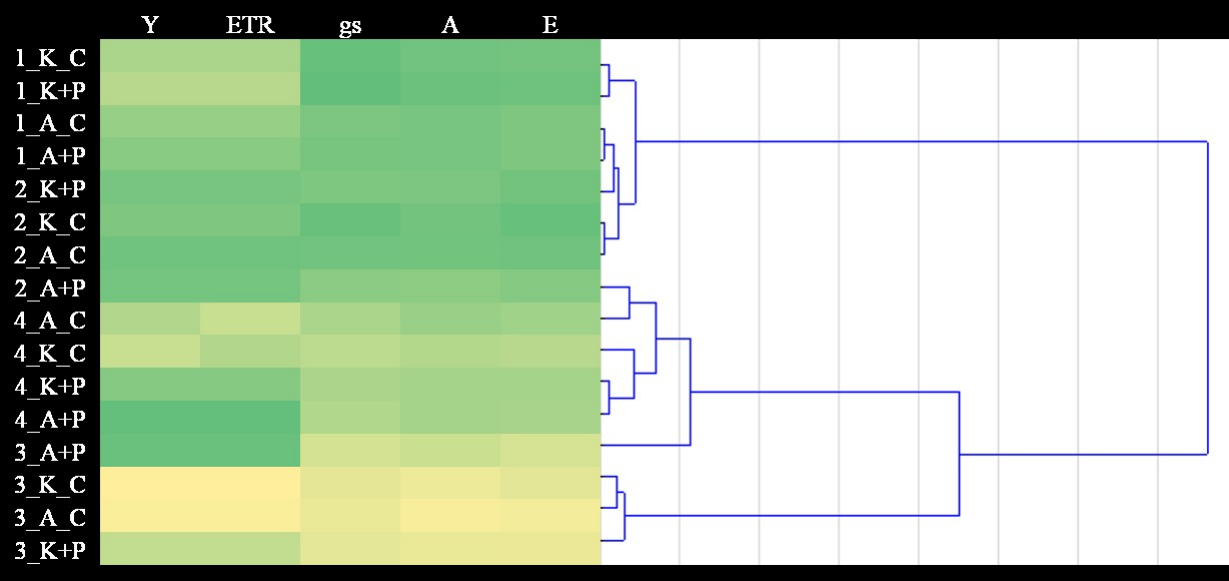
Comparable reaction between plant photosynthesis and chlorophyll fluorescence parameters in conventional and stay green maize varieties and the use of preparation in the fourth measurement phase. Parameters: Quantum Yield of PSII (Y), Electron Transport Rate (ETR), Stomatal conductance of H_2_O (gs; mmol m^-2^ s^-1^), Transpiration rate (E; mmol m^-2^ s^-1^ at a light intensity of 400 μmol photon m^-2^ s^-1^), Photosynthetic rate (A; μmol CO_2_ m^-2^ s^-1^ at a light intensity of 400 μmol photon m^-2^ s^-1^), Measurements: 1- first measurement, 2- spraying, 3- drought stress, 4- regeneration. Objects: ‘KWS 1325’_ control (K_C), ‘Ambrosini’ _ control (A_C), ‘KWS 1325’ + preparation (K+P), ‘Ambrosini’ + preparation (A+P).

The heatmap of interactions between the mean values of plant photosynthesis and chlorophyll fluorescence parameters revealed similarities between ‘Ambrosini’ plants treated with the preparation during drought stress and both varieties after regeneration. The remainder of the varieties in the drought stress phase were grouped together. A heatmap was used to show the lowest and highest parameter values. The highest mean quantum Yield PSII and ETR values were recorded in the ‘Ambrosini’ plants treated with the preparation during the drought stress phase. Moreover, the highest values of gs, A and E were found in the first measurement before stress was induced in ‘KWS 1325’on control and with the use of preparation. The highest parameter values associated with plant photosynthesis were observed, together with high chlorophyll fluorescence values, in the ‘Ambrosini’ and ‘KWS 1325’ control plants before the induction of drought stress (Fig 2).

## Discussion

Maize is a sensitive crop and droughts can lead to severe losses. However, studies indicate that the reaction of a plant to drought stress differs depending on the scale of the damage, the period of time that the stress occurs, the phenological stage, or the genetic background of the plant [28]. Responses to drought are multiple and interconnected, and they impair numerous metabolic and physiological processes in plants [29]. Many studies have shown that such reactions lead to limitations in plant growth, decrease in the content of chlorophyll pigments and water, and generally reduce the photosynthetic efficiency of plants [30,31,32,33,34,35,36]. Previous studies have also shown that plants react differently to drought stress depending on the genotype [37,38].

In our study, the varieties showed different responses to the application of the pyraclostrobin + epoxiconazole fungicide preparation, although their reaction to drought stress was similar. The ‘Ambrosini’ plants exhibited defensive reactions to stress as early as 48 hours after spraying with the preparation despite showing no real stress effects. This was seen by the lack of complete closure of the stomata (high gs), active transpiration rates (E), and a more efficient photosynthetic rate (A). This may have been associated by an early “switching on” of the defence mechanisms of the plants in order to prepare them for future stress. The ‘Ambrosini’ plants subjected to the protective treatment with the preparation endured drought stress better than the ‘KWS 1325’ plants. In the case of the latter, no defensive mechanism was observed. The mechanism described above protected the plants subjected to drought stress to some extent. Similar studies on the application of bio-stimulators in oil seed rape, some ornamental and vegetable species observed no effect of treatment [13] or that the treatment negatively affected the plants [15,16].

In the ‘KWS 1325’ plants, no significant difference in A was observed during drought stress in the preparation-treated plants when compared to the control groups. At lower light intensity values, the ‘KWS 1325’ variety exhibited more intense photosynthesis rates (Ec and the theoretical point of light saturation). Thus, this variety adjusted better to the conditions of low light intensity and showed a better ability to regenerate. The ‘Ambrosini’ variety, in contrast, showed a lesser ability to use low light intensity.

The tested varieties displayed different reactions to the application of the preparation. In the ‘KWS 1325’ plants, no direct reaction was observed after spraying, and the application of the preparation did not facilitate photosynthesis or increase the transpiration rate. Notably, the regeneration process was strongly enhanced by the use of the preparation in those plants. As a result, the mass gain of the ‘KWS 1325’ plants was similar to that of the ‘Ambrosini’ plants.

Chaves *et al*. [39] and Mahajan and Tuteja [40] have suggested that the first reaction to drought stress is a narrowing of the stomata in plant leaves (as a plant defence mechanism), which results in a decrease in the rate of photosynthesis and limits gas exchange and CO_2_ uptake. In turn, this leads to a reduction in the formation of adenosine triphosphate (ATP) and nicotinamide adenine dinucleotide phosphate (NADPH), which is particularly important under stress conditions. These limitations were related to the rate of electron transport through the imbalance between the photochemical activity of PSII and the demand for NADPH as a result of the reduction in CO_2_ assimilation.

Maize shows variable sensitivity to drought at some critical periods: the productivity stage [41], or in bracketing flowering [42]. Water deficit during these periods can lead to a severe loss in maize yield and its components. Nejad *et al*. [43] reported that water stress induced through, during and after the flowering phases decreased corn yield by 21%, 5%, 25%, respectively, in comparison to control plants. It has also been reported that the decrease in grain yield due to severe stress can reach up to 80% [41,44,45].

In this work, we did not focus on the reduction of yield due to drought stress but were more interested in plant protection and the preservation of productivity under drought stress. The use of a preparation that supports plants under drought stress produced the desired effect, which differed between the two maize varieties. After the experiment, the dry matter weight of both varieties and the buds of the ‘Ambrosini’ cobs increased by 1.04 g per plant, respectively. For the ‘KWS 1325’ plants, the increase was 0.93 g per plant, respectively. The ‘Ambrosini’ plants were taller and accumulated more dry matter, especially in their cob buds, when compared with the ‘KWS 1325’ plants. An increase in plant biomass (6.99%) as a result of the use of the preparation was observed after plant regeneration. The ‘KWS 1325’ plant variety exhibited a greater increase than the ‘Ambrosini’ variety. The biomass of each part of the plant is the final result of the efficiency of physiological processes that occur in the plant during the vegetative phase. The preparation used in this experiment belongs to the strobilurin class of agricultural fungicides that were initially developed to control fungal disease but also show growth promoting and yield enhancing qualities under field conditions. Swoboda and Pedersen [46] also reported that growth of soybean was enhanced by a foliar spray of pyraclostrobin. Some effects in boosting yields have also been shown by Nelson and Meinhardt [47], Henry *et al*. [48] and Hill *et al*. [49] in wheat, corn, soybean and other crops.

The inhibition of photosynthesis is known as the first physiological result of drought stress [50]. As a period of drought increases, the photosynthetic rate of the plants continues to decrease and is the main factor for the subsequent reduction of yield [51,52]. Our study is in agreement with previous studies that observed that assimilation, transfer and use of light energy decreased in maize varieties under drought conditions.

In the ‘Ambrosini’ variety, the use of the preparation boosted both photosynthesis and transpiration rates under drought conditions. As such, the regeneration process was promoted to some extent as those plants were less stressed. The stomata in the ‘Ambrosini’ plants were opened more widely. Other studies have confirmed that following stress induced by moisture deficits in soils, plants close the stomata in the leaves, decrease rates of net CO_2_ uptake and adjust their metabolic processes [23,53]. The mode of action of the preparation used here is preventative, so the strobilurin fungicide group are called new generation fungicides with a broad spectrum of activity. It has been shown that they change metabolism pathways and regulate phytohormonal levels to overcome stress. As a result, plants achieve greater biomass production and yields [54]. Köhle *et al*. [55] indicate that pyraclostrobin induces the activity of ACC-synthase and synthesis of ethylene in wheat during stress and plant senescence. The same authors point out that strobilurin breaks down to the L-tryptophan, a natural precursor of indole-3-acetic acid (IAA) and alleviates oxidative stress increase in the activity of antioxidative enzymes, such as superoxide-dismutases, catalases and peroxidases in wheat plants. Thus, wheat treated with pyraclostrobin in some experiments showed a doubling of enzymatic activity, and as a result the plants become more tolerant to stress.

During continuous drought, plants attempt to protect themselves against transpiration by closing their stomata [56]. There is general agreement that a decrease in the photosynthesis rate under water stress can result from both stomatal and non-stomatal restrictions [31]. However, the stomata in some plants remain open despite the loss of water and higher transpiration rates. Plants can also expel water from their cuticles; this feature depends on the species and their ability to conduct water from the cuticle [57]. The non-stomatal mechanisms are characterised by disturbances in the processes of accumulation, transport, and distribution of assimilates, which in effect causes changes in chlorophyll synthesis or functional and structural changes in chloroplasts [58]. The use of the preparation increased gs and the intensity of photosynthesis in the ‘Ambrosini’ plants. The increased concentration of intracellular CO_2_ suggests greater respiration rates and thus a greater consumption of energy and could explain why the dry matter content of the ‘Ambrosini’ plants was lower after regeneration. In both varieties, the use of the preparation increased respiration rates, although the increase in respiration in the ‘Ambrosini’ plants was observed to be much higher. In similar experiments, the application of pyraclostrobin + epoxiconazole fungicides in maize did not cause changes in stomatal conductance, although the authors suggested that the plants were not under stress [59]. Furthermore, the authors indicated that using pyraclostrobin + epoxiconazole at the recommended dose did not cause toxic effects [59].

The mechanisms of dissipation and photoprotection in the two maize varieties in our study were different. This study revealed varietal differences in the response to drought stress, which is consistent with the results obtained by Liu *et al*. [60], who considered that a decrease in photosynthesis and transpiration rates can be attributed not only to gs but also to other mechanisms in the elongation stage of maize. One possibility to protect the maize leaf from light-induced damage under drought stress is an increase in exchanged excessive energy in the form of thermal energy when maintained under a high light intensity.

In the literature, cultivars that are resistant or show more tolerance to stress have been shown to have a greater chlorophyll potential yield [61,62,63]. Our results confirmed the relationship between the chlorophyll content in the leaves and resistance to drought. The ‘Ambrosini’ variety contained more chlorophyll in its leaves and was more resistant to drought stress than the ‘KWS 1325’ variety. In contrast to the beginning of the experiment, plants contained a higher concentration of chlorophyll after drought. The preparation had a positive impact on the chlorophyll content in the maize leaves. This result can be related to the mode of action of the preparation, e.g. pyraclostrobin is known to retard senescence. In an experiment with wheat, strobilurin reduced the loss of chlorophyll (a parameter used to determine senescence) and the effect was preceded by inhibition of ethylene formation [64]. It is suggested that an increase in the photosynthetic period increases the quantity of assimilate available for grain filling. As strobilurin fungicides cause the photosynthetic active leaf area to stay green for longer, this may be the main factor in increased yields [65].

In this study, both varieties were protected by the preparation, but differed in their reaction to drought stress. The yield of PSII (expressed by the Yield parameter) was 13.1% higher in the ‘Ambrosini’ plants than in the ‘KWS 1325’ plants (Fig 1A). The measurement was done during the drought stress phase. Moreover, ETR was also higher in the ‘Ambrosini ‘plants; 13.3% greater than in the ‘KWS 1325’ plants (Fig 1B). Furthermore, the parameter A and gs values were 2.1 times higher for the ‘Ambrosini’ plants than for the ‘KWS 1325’ plants. The measurement was carried out on plants protected with the preparation during the drought stress phase. Notably, the E value for the ‘Ambrosini’ plants was 78% higher than for the ‘KWS 1325’ plants (Fig 1D). Therefore, the use of the preparation on the ‘Ambrosini’ variety did not reduce transpiration rates despite drought stress. This is important in the case of C4 plants where WUE is twice as high as in C3 plants (where 1.3–2 g of dry matter production requires 1 kg of water). Consequently, plants need an enormous amount of water for growth [66], which can be compared to the amount of CO_2_ absorbed on a molar basis, although the amount of water transpired from leaves through their stomata was 500–1000 times higher [67]. The biggest challenge for crop growers is to increase the efficiency of water use as this is essential to maintaining yield levels, especially when drought conditions are forecast [68].

Chlorophyll molecules in a leaf absorb light energy, which results in various reactions. This energy can be used to conduct photosynthesis. The surplus energy can be dissipated as heat, or it can be re-emitted as light-chlorophyll fluorescence. These processes occur in competition. Any increase in the efficiency of one can result in a decrease in the yield of the other two [26]. It is well accepted that photoinhibition is one of the primary physiological consequences of drought stress [50,69]. Baker and Bowyer [70] indicated that alterations in PSII activity under water stress are related to photoinhibition rather than to any direct damage to PSII. However, non-stomatal limitations can occur with an increase in stress intensity. Such restrictions can involve an inhibition or damage to the biochemical metabolism and photochemical reactions (PSII activity) [71,72,73].

Recent studies have shown that chlorophyll fluorescence together with photosynthesis can better explain the differentiation that occurs in maize varieties. Expression of this diversity can be noticed in drought conditions especially if fluorescence and gas exchange are measured together [74,75]. To date, studies have showed a decrease in gs in all plants under stress conditions, although no difference has been observed between hybrids [76].

Recent studies have revealed an information transfer between roots and shoot during rehydration after drought stress, in the form of electric and hydraulic signals that could elicit subsequent physiological control of net CO_2_ uptake. Maize plants have evolutionary adapted to water-limited habitats by propagation of electric and hydraulic signals in the root system. Such signals allow plants to respond rapidly to increasing soil moisture, which favour C4 plants, such as maize [77].

## Conclusions

The use of pyraclostrobin in combination with epoxiconazole three days before inducing drought stress improved the efficiency of photosynthesis in the tested maize varieties. Plant photosynthetic efficiency (ETR and Yield parameters) during the drought stress phase and after regeneration was significantly higher than in the control plants. The ‘Ambrosini’ plants produced a significantly greater weight of cob buds, while the ‘KWS 1325’ variety produced higher whole plant biomass compared with the control plants. Plants treated with the preparation were found to be more resilient under drought stress than the control plants. In addition, the effectiveness of their regeneration was also better. The effects of the preparation were more clearly observed in the stay-green ‘Ambrosini’ variety than in the conventional ‘KWS 1325’ variety.

## Supporting information

S1 File Raw data. (XLSX)
